# *FADS* Polymorphisms Affect the Clinical and Biochemical Phenotypes of Metabolic Syndrome

**DOI:** 10.3390/metabo12060568

**Published:** 2022-06-20

**Authors:** Aleš Žák, Marie Jáchymová, Michal Burda, Barbora Staňková, Miroslav Zeman, Adolf Slabý, Marek Vecka, Ondřej Šeda

**Affiliations:** 1 4th Department of Medicine, 1st Medical Faculty, Charles University and the General University Hospital in Prague, 128 08 Prague, Czech Republic; zak.ales@email.cz (A.Ž.); barsta@atlas.cz (B.S.); miroslav.zeman@vfn.cz (M.Z.); adaslaby@seznam.cz (A.S.); 2 Institute of Clinical Chemistry and Laboratory Diagnostics, 1st Medical Faculty, Charles University and the General University Hospital in Prague, 128 08 Prague, Czech Republic; jachymova@yahoo.com; 3 Institute for Research and Applications of Fuzzy Modeling, University of Ostrava, 701 03 Ostrava, Czech Republic; michal.burda@osu.cz; 4 Institute of Biology and Medical Genetics, 1st Medical Faculty, Charles University and the General University Hospital in Prague, 128 00 Prague, Czech Republic; ondrej.seda@lf1.cuni.cz

**Keywords:** metabolic syndrome, fatty acid pattern, cluster analysis, single-nucleotide polymorphism, haplotypes, *FADS1*, *FADS2*

## Abstract

Long-chain polyunsaturated fatty acids (LC-PUFAs) play important roles in human health, from controlling inflammation to lipid and glucose homeostasis. In our previous study, which employed a cluster analysis of a plasma fatty acid (FA) pattern, we identified two clusters of metabolic syndrome (MetS) independent of clinical and biochemical parameters within the whole study group (controls together with metabolic syndrome (MetS) patients). FA desaturase (*FADS*) genes are the key regulators of LC-PUFA metabolism. The aim of this study was to analyze associations between *FADS* polymorphisms and clusters of MetS. The study group consisted of 188 controls and 166 patients with MetS. The first cluster contained 71 controls (CON1) and 109 MetS patients (MetS1). The second cluster consisted of 117 controls (CON2) and 57 MetS patients (MetS2). In comparison with MetS2, cluster MetS1 displayed a more adverse risk profile. Cluster CON1 had, in comparison with CON2, higher body weight and increased triacylglycerol levels (*p* < 0.05). We found that the *FADS* rs174537 (*p* < 0.001), rs174570 (*p* < 0.01), and rs174602 (*p* < 0.05) polymorphisms along with two inferred haplotypes had statistically significant genotype associations with the splitting of MetS into MetS1 and MetS2. Conversely, we observed no significant differences in the distribution of *FADS* polymorphisms between MetS and CON subjects, or between CON1 and CON2. These associations between *FADS* polymorphisms and two clusters of MetS (differing in waist circumference, HOMA-IR, lipolysis, and oxidative stress) implicate the important influence of genetic factors on the phenotypic manifestation of MetS.

## 1. Introduction

Metabolic syndrome (MetS) is a group of clinical, biochemical, and metabolic conditions that include abdominal obesity, insulin resistance, atherogenic dyslipidemia, and arterial hypertension, all known as MetS components. MetS is associated with a high risk of developing type 2 diabetes mellitus (T2DM), cardiovascular diseases (CVD), as well as a number of other clinical conditions, such as nonalcoholic fatty liver disease (NAFLD), vascular dementia, Alzheimer’s disease, and various tumors (especially pancreatic and colorectal cancer). The increasing prevalence of MetS, due to the obesity pandemic, represents a serious public health concern [1,2,3]. The pathophysiology of MetS involves several complex mechanisms, such as age, gender, and genetic factors [4,5,6]. Lifestyle (overeating and lack of physical activity) and the quantity and composition of dietary fat and carbohydrates (fructose and sucrose) consumed are also important contributors to MetS development [3,7].

In addition to the above components, MetS is characterized by increased levels of oxidative stress, low-grade inflammation, hormonal activation, endothelial dysfunction, and variations in the composition of esterified fatty acids (FAs) in various body compartments [3,7,8]. Like other insulin resistance states, MetS is mostly connected to increased proportions of serum saturated FAs (SFAs), palmitoleic acid (POA, 16:1n-7), and dihomo-γ-linolenic acid (DGLA, 20:3n-6), whereas proportions of linoleic acid (LA, 18:2n-6) and total n-6 polyunsaturated FAs (n-6 PUFA) are decreased. Moreover, MetS is linked to increased estimated activities of the enzymes delta-9 desaturase (D9D) and delta-6 desaturase (D6D), which catalyze the synthesis of POA from palmitic acid (PA, 16:0), oleic acid (OA, 18:1n-9) from stearic acid (SA, 18:0), and γ-linolenic acid (GLA, 18:3n-6) from LA. In contrast, the activity of delta-5 desaturase (D5D), which catalyzes the synthesis of arachidonic acid (AA, 20:4n-6) from DGLA is decreased. The dysregulated FA metabolism and composition are connected to an altered n-3 PUFA/n-6 PUFA ratio [6,9,10,11,12]. Similar FA alterations correlate with both the total and cardiovascular mortality and the risk of developing T2DM.

MetS and other cardiometabolic diseases such as obesity, T2DM, NAFLD, and CVD, are characterized by decreased concentrations of long-chain polyunsaturated fatty acids (LC-PUFA) in plasma and body tissues [7,13,14]. The biological effects of LC-PUFAs are supposed to be mediated by the availability of PUFAs with ≥20 carbons and ≥3 double bonds [15].

LC-PUFAs have a wide range of regulatory, autocrine, and paracrine effects. LC-PUFAs are important components of cell membranes, which are known to affect fluidity and permeability, thus, influencing the functions of membrane-associated proteins. They also act as substrates for the synthesis of eicosanoids and docosanoids (being substrates for the synthesis of specialized proresolving mediators) and, thus, control inflammation, act as signaling molecules (eicosanoids and endocannabinoids), and regulate gene expression by serving as ligands to transcription factors such as peroxisome proliferator-activated receptors γ/α, (PPARγ/α), nuclear factor kappa B (NFκB), and sterol regulatory element-binding protein-1 (SREBP-1), all of which affect the lipid and glucose metabolism [6,16,17].

The FA composition of plasma lipids and cell membranes depends on their dietary intake and endogenous FA metabolism, which are in turn influenced by many factors, including age, gender, ethnicity, health status, and, importantly, genetics [18]. Apart from the dietary supply of preformed LC-PUFAs, they can be derived from precursor essential FAs, LA, and α-linolenic acid (ALA, 18:3n-3) via successive desaturation and elongation. The activities of fatty acid desaturases (*FADSs*) and elongases significantly affect FA profiles in different body compartments. Delta-5 desaturase (D5D, *FADS1*) and delta-6 desaturase (D6D, *FADS2*) are the key enzymes required for the synthesis of LC-PUFA [19,20]. D5D and D6D catalyze the conversion of n-6 and n-3 PUFAs to their longer and more unsaturated products. Through a series of desaturation and elongation reactions (mainly in the liver), the parent n-6 PUFA, LA, is converted to AA, while the parent n-3 PUFA, ALA, is converted to eicosapentaenoic acid (EPA, 20:5n-3) and docosahexaenoic acid (DHA, 22:6n-3) [13,14,16].

Both D5D and D6D are encoded by the fatty acid desaturase 1 (*FADS1*) and *FADS2* genes, which are mapped on human chromosome 11q12.2-13.1. Activities of D5D and D6D are associated with the LC-PUFA content in the body compartments and are related to MetS risk. Candidate gene and genome-wide association studies (GWASs) have described associations between minor alleles of single-nucleotide polymorphisms (SNPs) in the *FADS1*–*FADS2* genes and lower concentrations of LC-PUFAs. Carriers of the minor alleles exhibit increased concentrations of desaturase substrates (LA and ALA) and decreased levels of desaturase products (AA, EPA, and DHA) [14,21]. Only in the *FADS2* rs968567 (C > T) polymorphism has a minor allele been shown to have higher promoter activity of D6D [22]. GWASs have revealed the presence of two common *FADS* haplotypes (A and D), which differentially regulate LC-PUFA synthesis. While haplotype D is associated with an increased ability to synthesize AA and EPA, haplotype A is associated with an opposite synthesizing effect on these LC-PUFAs. Variants of *FADS1*/*FADS2* genes (as well as the statistically reconstructed haplotypes derived from them) show the strongest association with AA, but also with LA, ALA, eicosadienoic acid (20:2n-6), DGLA, docosapentaenoic acid (DPA, 20:5n-3), and DHA (22:6n-3) [23,24,25].

SNPs of *FADS1*–*FADS2* genes have been strongly associated with various metabolic phenotypes/traits, including obesity, T2DM, dyslipidemia, and complex conditions, such as coronary artery disease (CAD), ischemic stroke, and NAFLD [18,26,27,28].

Some studies have found significant genotype–phenotype associations between variants of *FADS1*/*FADS2* (rs174547, rs174575) genes and atherogenic dyslipidemia (high triacylglycerol (TAG) and low high-density lipoprotein cholesterol (HDL-C) levels) [29,30,31,32,33,34]. The *FADS1* variant (rs174547) is associated with an increased risk of developing MetS [32]. Chinese authors found a significant association between *FADS1* rs174547 and MetS, as well as its components (waist circumference, blood pressure, and a low HDL-C level) [34]. In diabetics, the minor allele of the *FADS2* variant (rs174575) associated with a higher plasma total cholesterol, LDL-C, and TAG was reported [35]. In addition, several *FADS* polymorphisms (rs174537, rs174575, rs174547, rs174576, rs174616, and rs174550) have been related to lipoprotein particle size obesity and the abdominal distribution of adipose tissue and low-grade inflammation [36,37,38,39,40,41]. Variants (rs174537, rs174575) of the *FADS1*/*FADS2* genes are also associated with a higher oxidative stress level [21,37,40,42]. Some studies have found associations between *FADS1*/*FADS2* variants (rs174546, rs174550, rs174537, rs174575, and rs174570) and insulin sensitivity, the serum glucose level, and risk of T2DM [43,44,45], while others have linked *FADS1*/*FADS2* variants (rs1535, rs174547, rs174546, rs174537, rs174549, and rs9957425) to increased blood pressure (arterial hypertension, respectively) and obesity [46,47,48,49,50]. Associations between *FADS1*/*FADS2* variants and complex conditions such as MetS, T2DM, and cardiovascular disease (e.g., acute coronary syndromes, CAD, and ischemic stroke) have likewise been described. An alternative/minor allele of *FADS1* rs174537 was linked to T2DM in an Iranian population [44], and *FADS1* rs74556 and *FADS2* rs174617 have been linked with acute coronary syndromes [51]. The GG genotype of the *FADS1* variant rs174537 increases the risk of developing T2DM and coronary artery disease [52]. T alleles and TT genotypes of the *FADS1*/*FADS2* variants are associated with coronary artery disease and ischemic stroke. The minor allele of rs174547 is connected to a lower risk of ischemic stroke [28,53]. The *FADS1* (rs174537) polymorphism, which is associated with an increased GG genotype frequency in Afro-Americans, is supposed to be connected to high levels of AA, leading to an increased level of low-grade inflammation and a greater risk of developing T2DM [54].

An increased concentration of ALA in adipose tissue in combination with an *FADS2* SNP (rs3834458 T/-) has been implicated in the development of MetS via a decreased conversion of ALA to EPA [55]. Finally, the elevated activity of aggregate delta desaturase activity represents an independent risk factor for ischemic heart disease. Aggregate desaturase activity has been associated with risk alleles of *FADS1*/*FADS2*, which condition the proinflammatory PUFA profile in correlation with hs-CRP levels [56].

In our previous study using a cluster analysis of plasma FA patterns, we described two clusters (or phenotypes) of MetS, independent of clinical and biochemical parameters. In comparison with cluster 2 (MetS2), cluster 1 (MetS1) displayed a greater consistency and more adverse risk [9].

The aim of the present study was to analyze associations between selected polymorphisms of the *FADS1*-*FADS2* genes (including reconstructed haplotypes) and phenotypes (or clusters) of MetS patients and controls (CON) in a Czech population.

## 2. Results

### 2.1. Clinical and Biochemical Parameters

Patients with MetS significantly differed from CON subjects in all clinical (body mass index, blood pressure, waist circumference, and fat mass) and biochemical (TC, TAG, HDL-C, apoB, glucose, CD-LDL, insulin, and HOMA-IR) parameters consistent with the MetS phenotype (Table 1).

Based on the cluster analysis that included six selected FA patterns obtained from a linear discriminant analysis, two clusters were found in the whole study group (CON and MetS groups together). In the MetS group, 109 patients (65.7%) were classified as cluster 1 (MetS1) and 57 patients (34.3%) were classified as cluster 2 (MetS2). Conversely, in the control group, 71 probands (37.8%) were classified as cluster 1 (CON1) and 117 subjects (62.2%) were classified as cluster 2 (CON2). These results indicated a nonrandom distribution of subjects in both groups into cluster 1 and cluster 2 (χ^2^ = 26.35; *p* < 0.001). This means that MetS was present mainly in cluster 1, while the CON probands presented mainly in cluster 2. Patients with MetS in cluster 1 (MetS1) had a more adverse metabolic profile in comparison with patients in cluster 2 (MetS2). There were no statistically significant differences between both clusters with regard to the sex ratio, age, BMI, fat mass, systolic and diastolic blood pressure, total cholesterol, HDL-C, triglycerides, or apoB concentrations. Characteristically, patients with MetS1 exhibited an increased waist circumference and HOMA-IR (both *p* < 0.05), as well as increased glucose (*p* < 0.01), NEFA (*p* < 0.001), and CD-LDL concentrations (*p* < 0.05) (Table 2).

After splitting the CON group according to the results of the cluster analysis, individuals in CON1 were found to have an increased body weight (by 9%; *p* < 0.01), BMI (by 5.5%, *p* < 0.05), and TAG (by 23%, *p* < 0.01). After the ANCOVA adjustment with body weight as the covariate, the only parameter to increase in CON1 probands compared with CON2 was the TAG level (*p* < 0.05). However, the median difference between CON1 and CON2 (0.3 mmol/L) proved clinically insignificant (Table 3).

Differences in clinical and biochemical traits between MetS1 and CON1 followed a similar pattern to the differences between MetS and CON. However, after adjusting for body weight, the differences in TC and apoB levels between MetS1 and CON1 proved nonsignificant (Supplementary Table S1).

Patients in Met2, compared to CON2 individuals, had significantly changed nearly all studied traits except for TC, apoB, NEFA, and CD-LDL levels (Supplementary Table S2).

### 2.2. Fatty Acid Profiles in Plasma Phospholipids

The FA compositions of plasma phospholipids (PL) for MetS and CON are given in Table 4. In MetS patients, we found significantly increased concentrations of POA (*p* < 0.01), SA (*p* < 0.001), DGLA (*p* < 0.0001), and decreased levels of LA (*p* < 0.001). There was an increased sum of saturated FA (ΣSFA) (*p* < 0.0001) and decreased Σn-6 PUFA (*p* < 0.001) in MetS compared to the CON group. These changes were accompanied by increased activities of D9D and D6D (both *p* < 0.05) and decreased activity of D5D (*p* < 0.01).

A comparison of FA patterns between patients in the MetS1 and MetS2 clusters is given in Table 5. In MetS patients, we found increased concentrations of POA (*p* < 0.0001), OA (*p* < 0.0001), and vaccenic acid (VA, 18:1n:7, *p* < 0.01), and opposite changes in the level of LA (*p* < 0.0001). Moreover, MetS1 patients exhibited increased levels of precursors of proinflammatory eicosanoids, such as GLA (*p* < 0.05), DGLA (*p* < 0.05), and AA (*p* < 0.05). MetS1 patients also displayed statistically increased levels of n-3 PUFA, such as eicosapentaenoic acid (EPA, 20:5n-3, *p* < 0.0001), docosapentaenoic acid (DPA, 22:5n-3, *p* < 0.0001), and docosahexaenoic acid (DHA, 22:6n-3, *p* < 0.0001). MetS1 exhibited increases in ΣSFA, the Σ of monounsaturated (ΣMFA), and Σ n-3 PUFA (all *p* < 0.0001), as well as in the activities of D9D and D6D (both *p* < 0.0001).

The FA compositions of CON1 and CON2 participants are given in Supplementary Table S3. Changes in FA composition were similar to those observed for the MetS1 and MetS2 clusters. Participants in CON1 had significantly increased levels of PA (*p* < 0.01), POA (16:1n-7, *p* < 0.000), SA (*p* < 0.01), and OA (*p* < 0.05), as well as higher levels of n-6 LC-PUFA (DGLA and AA, both *p* < 0.001) and n-3 LC-PUFA (EPA, DPA, and DHA, all *p* < 0.001). Similarly, levels of ΣSFA, ΣMFA, Σn-3PUFA, and Σn-6PUFA significantly increased (all *p* < 0.001), as did the activities of D9D and D6D (both *p* < 0.0001). A comparison of FA patterns and derived parameters between the MetS1 and CON1 groups is given in Supplementary Table S4. MetS1 patients only exhibited a decreased activity of D5D (*p* < 0.05). Differences in FA composition between MetS2 patients and CON2 probands are shown in Supplementary Table S5. Patients in MetS1 had decreased OA (*p* < 0.05) and VA (both *p* < 0.01) levels, which were associated with mild changes in ΣSFA and ΣMFA levels (both *p* < 0.05).

### 2.3. Genetic Analyses and Statistically Reconstructed Haplotypes

**Figure 1:**
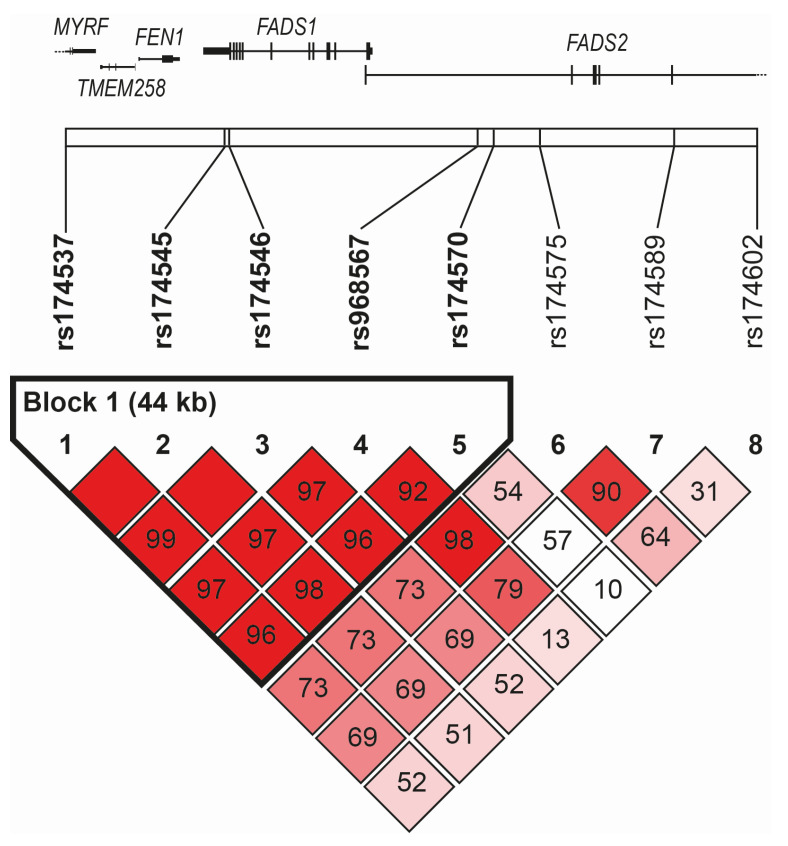
Linkage disequilibrium among studied SNPs of *FADS1*–*FADS2* region.

In our study, three *FADS1* SNPs (rs174537, rs174545, and rs174546) and five *FADS2* SNPs (rs174570, rs174575, rs174602, rs174589, and rs968567) were analyzed. All studied variants of the *FADS1*–*FADS2* genes were in Hardy–Weinberg equilibrium (*p* > 0.05). The polymorphism *FADS1* rs174537 was in the near-complete linkage disequilibrium (LD) (r^2^ = 0.97–0.99) with the two other *FADS1* SNPs (rs174545 and rs174546) and two of the *FADS2* SNPs (rs968567 and rs174570) (see Figure 1).

The linkage disequilibrium plot based on D’ spanned the genomic region of chromosome 11, including the *FADS1* and *FADS2* genes (chr11: 61,785,208–61,856,942, genome assembly GRCh38.p14) with the indication of the position of single-nucleotide polymorphisms genotyped in the current study. The definition of blocks was computed using the Genotype Resolution and Block Identification using Likelihood (GERBIL) algorithm implemented in the Genotype Visualization and Algorithmic Tool (GEVALT) software, version 2. Numbers indicated pair-wise D’ values, and the color-coding of boxes was based on D’/LOD: shades of pink/red for LOD > 2, D’ < 1 (bright red for D’ = 1), white for LOD < 2, D’< 1.

The frequencies of reference (major) and alternative (minor) alleles of all polymorphisms analyzed in this study were not significantly different from frequencies found in other European populations (Supplementary Table S6).

The numbers and frequencies of allele and genotype variants in the *FADS1*–*FADS2* genes across studied groups based on the cluster analysis are given in Table 6 and Table 7. The differences in allele and genotype frequencies were not statistically significant for MetS vs. CON, CON1 vs. CON2, MetS1 vs. CON1, and MetS2 vs. CON2 in any of the analyzed variants in the *FADS1*/*FADS2* genes.

Statistically significant differences between MetS1 and Mets2 were only found for the *FADS1* variants rs174537, rs174545, and rs174546 (*p* = 0.0024 for both allele and genotype), *FADS2* rs174570 (*p* = 0.014 for genotype, *p* = 0.009 for allele), and *FADS2* rs174602 (*p* = 0.048 for genotype, *p* = 0.0248 for allele) (see Table 7).

After simultaneous block partitioning and haplotype phasing, a single haplotype block (rs174537-rs174545-rs174546-rs968567-rs174570) was revealed within the region spanned by the genotyped SNPs (Figure 1). Using five-SNP haplotypes, we modeled four haplotypes with an estimated frequency greater than 1%. The frequencies of individual haplotypes were GGGCC (66.2%) > TCATC (14.9%) > TCACT (13.0%) > TCACC (4.9%). The association of haplotypes with the individual clusters showed a significant result only when comparing MetS1 vs. MetS2 clusters, corroborating the individual SNP associations. After one million permutations, the corrected *p*-values obtained were 0.0002 and 0.0027 for the GGGCC and TCACT haplotypes, respectively.

## 3. Discussion

The development of MetS, such as other cardiometabolic diseases, is influenced both by lifestyle and genetic factors. An increased energy intake, particularly saturated fat along with an imbalance in the type of dietary fat consumed, results in obesity, insulin resistance, and ectopic fat accumulation. These states are characterized by decreased concentrations of LC-PUFA in body tissues. The individual components of MetS are variably influenced by SFA, monounsaturated FA (MFA), and LC-PUFA of the n-3 and n-6 series. These fatty acids are understood to have effects on plasma lipids/lipoproteins, blood pressure, and insulin secretion, as well as on low-grade inflammation and oxidative stress [13,14,16,26]. LC-PUFAs play an important protective role by controlling the synthesis and oxidation of SFA and MFA, decreasing the hepatic fat content and improving blood lipid profiles associated with cardiovascular risk [17,57].

The availability of LC-PUFA in body tissues depends on the dietary intake as well as on endogenous factors influencing the FA metabolism. Recently, attention has been focused on fatty acid desaturases and elongases. *FADS1* and *FADS2* encode the enzymes D5D and D6D, respectively [14,18,58]. These are the key enzymes in the conversion of n-6 and n-3 PUFA to their longer and more unsaturated products.

Activities of D5D and D6D depend on the genetic background and on other factors, especially nutritional, hormonal, and environmental. The expression of D5D is increased by insulin and inhibited by a fat-free diet, exogenous cholesterol, trans-MFA, n-3 PUFA, glucagon, adrenalin, and glucocorticoids. The expression of D6D increases the deficiency of essential FA (EFA) (e.g., LA and ALA), Zn, Mg, and pyridoxine. The Inhibition of D6D is associated with fasting, ethanol, dietary SFA, cholesterol, PUFA (of both n-3 and n-6 series), glucagon, glucocorticoids, and thyroxine [59].

*FADS1* and *FADS2* polymorphisms are considered the most important factors contributing to variability in LC-PUFA levels in plasma phospholipid and erythrocyte membranes. A variation in FA concentrations attributable to the SNPs of *FADS1*/*FADS2* genes amounts to 28% for AA and 10% for precursor FAs [16]. According to data on erythrocyte FA from the Framingham Offspring Study, a strong association has been shown between the *FADS* and *ELOVL* regions. SNPs in these regions accounted for a 8–14% variation in three FAs (AA, LA, and OA) and a 1–4% variation in another four FAs (ALA, DTA, GLA, and DPA). Polymorphisms of *FADS* and *ELOVL* revealed a 53% variance in DGLA levels [60].

In our study, we performed a cluster analysis that included six selected FAs (DGLA (20:3n-6), SA (18:0), myristic acid (MA, 14:0), DHA (22:6n-3), DPA (22:5n-3), and LA (18:2n-6)), revealing two clusters each in the CON and MetS groups. In the MetS group, 65.7% of patients were classified as phenotype/cluster 1 (MetS1) and 34.3% of patients were classified as MetS2. In the CON group, 37.8% of probands were classified as cluster 1 (CON1) and 62.2% of subjects were classified as CON2. These results indicated a nonrandom distribution of subjects in both groups into cluster 1 and cluster 2 in both groups, supporting the premise that two genotypes/clusters of MetS exist that are independent of clinical and laboratory parameters [9]. To our knowledge, this is the first phenotyping of MetS based on a cluster analysis of plasma FAs.

Two of these six FAs, namely, MA and SA, could be interpreted as surrogate markers of dietary SFA intake [59,61]. The remaining four FAs are important substrates, intermediates, and products of the LC-PUFA metabolism [21,58]. POA, OA, and VA levels, as well as the activities of D9D (SCD1, respectively) are supposed to be surrogate markers of de novo lipogenesis [62,63,64].

As expected, patients in the MetS1 cluster exhibited an increased waist circumference, a surrogate marker of abdominal obesity, and increased HOMA-IR. They also had increased plasma concentrations of NEFA and CD-LDL. In comparison with MetS2, MetS1 displayed a more adverse risk profile caused by a higher insulin resistance and an increased level of oxidative stress.

In comparison with CON2, patients in the CON1 cluster had a significantly increased body weight, waist circumference, and plasma TAG. After the ANCOVA adjustment with body weight as the covariate, the only parameter that remained significantly increased was the TAG level. The median difference between CON1 and CON2 (0.3 mmol/L) was clinically insignificant. Abdominal obesity is one of the major metabolic stressors leading to increased TAG levels. The persistence of elevated TAG levels after adjusting for body weight implicated the association of non-*FADS* genetic factors in the pathogenesis of mild hypertriglyceridemia. The elevation of TAG may be caused by the polygenic contribution of multiple risk alleles in genes that influence both VLDL production and removal [65]. Additionally, heterozygous carriers of the gene mutations that in a homozygous constitution cause chylomicronemia (e.g., LPL, APOC2, APOA5, LMF1, GPIHBP1, and GPD1) commonly display only moderately elevated serum TAG levels [29,66].

Our findings of higher HOMA-IR values and increased plasma concentrations of NEFA and CD-LDL in MetS concurred with our previous study and with the studies of other authors. A higher severity of MetS (as expressed by the number of MetS components) is associated with increased levels of plasma CD-LDL [67]. One study found that subjects with MetS had lower activities of plasma catalase and paraoxonase 1 [8]. Both CD-LDL and ox-LDL reflect systemic oxidative stress. CD-LDL is an indicator of minimally oxidized LDL (containing only oxidatively modified lipids), whereas ox-LDL contains both oxidatively modified lipids and proteins [68,69].

Abdominal obesity, insulin resistance, and T2DM risk are strongly associated with variants of the *FADS* gene cluster [70]. For instance, the gene–diet interaction was proved in the METSIM study. Carriers of the minor allele genotype (CC) for rs174550 (*FADS1*) had a lower plasma glucose concentration after LA consumption [21]. Moreover, an association between *FADS* genetic variants and disturbed glucose metabolism has been reported [71]. The rs174537 *FADS1* polymorphism, which is characterized by a “high-converting” GG genotype (being more frequent in African Americans), is supposed to be connected with high levels of AA and, thus, with a higher level of low-grade inflammation and with an increased risk of T2DM [54]. Finally, elevated aggregated delta desaturase activity represents an independent risk factor for CAD. Aggregated desaturase activity (AA/LA ratio) has been linked to risk alleles of *FADS1*/*FADS2*, which condition a proinflammatory PUFA profile and are correlated with hs-CRP levels [56]. In a large Italian population sample of obese children, a common variant in the gene *FADS1* (rs1535) has been associated with BMI [48]. SNPs of *FADS2* (rs174593, rs174616, and rs175576) have been documented to play a role in the interaction of polyphenols with PUFA levels and parameters of obesity [72], while lower levels of TC, LDL-C, and HDL-C have been associated with the minor allele of *FADS1* rs174547 [73]. Regarding the incidence of DM2T, one study found a positive association between D6D activity and *FADS1* rs174550, but, on the other hand, a clear inverse relation between the same SNP and D5D activity [43].

When comparing FA composition between the MetS and CON groups in our study, we found that MetS displayed increased levels of POA (16:1n-7), DGLA (20:3n-6), ∑SFA, and decreased levels of LA (18:2n-6) as well as ∑ n-6 PUFA. In comparison with MetS2, MetS1 showed an increased content of OA (18:1n-9), VA (18:1n-7), GLA (18:3n-6), AA (20:4n-6), EPA (20:5n-3), DPA (22:5n-3), ∑MFA, and ∑ n-3 PUFA, together with decreased levels of ∑ n-6 PUFA. MetS (compared to CON) and MetS1 (compared to MetS2) exhibited increased D6D and D9D (for PA) activities. In comparison with CON, MetS exhibited decreased activity of D5D. Our most distinct findings with regard to MetS were increased markers of D9D (SCD1) and D6D (*FADS2*) activities and decreased levels of LA. We observed a decrease in D5D (*FADS1*) only when comparing MetS and CON. The increased values of POA, D9D, and D6D we document here corresponded with previous findings by other authors [10,12,74]. A decrease in LA levels can be caused by several factors, including decreased dietary intake, increased peroxidation and β-oxidation, and a higher conversion of LA to proinflammatory eicosanoids [59].

The main result of our study was the statistically significant genotype associations of *FADS1* SNPs (rs174537, rs174545, and rs174546; all *p <* 0.01), *FADS2* SNPs (rs174570 and 174602, both *p <* 0.05), and two inferred haplotypes (GGGCC (*p <* 0.01) and TCACT (*p <* 0.05)) with the splitting of MetS into two phenotypes (MetS1 and MetS2). Additionally, the rs174537 polymorphism was in the near complete LD (r^2^ = 0.97–0.99) with the other two *FADS1* SNPs, rs174545 and rs174546, and with two of the *FADS2* SNPs, rs968567 and rs174570.

Our observation of a linkage disequilibrium block encompassing the *FADS1* gene and the adjacent part of the *FADS2* gene corresponded to previous findings by several European population studies investigating the extent LD blocks and associates with distinct FA profiles [17,23,25]. While rs174537 actually lies in the intron of the myelin regulatory factor gene with no reported function related to MetS features, the associations reported in this and previous studies (98 significant rs174537 associations are listed in the NHGRI-EBI catalog of human genome-wide association studies [75]) most likely reflect the near-complete linkage disequilibrium with other *FADS1* variants.

In comparison with MetS2, the MetS1 cluster/phenotype was characterized by a higher proportion of major alleles and genotypes of a homozygous constitution across all *FADS1* and *FADS2* SNPs significantly associated with the splitting of MetS into two phenotypes.

In comparison with MetS2, MetS1 exhibited increased D6D (*FADS2*) activity and aggregate desaturase activity (results are not given). We observed no significant differences in D5D (*FADS1*) between the two MetS phenotypes.

A cross-sectional Chinese study found a significant association between *FADS1* rs174547 (and a high LD linkage disequilibrium with rs174546 and rs174537) and MetS and its components. Carriers of the major allele and the TT genotype had a larger waist circumference (a surrogate marker of abdominal obesity), increased plasma glucose, higher blood pressure, and lower HDL-C [34]. Another Chinese study found that plasma FAs, delta desaturase activities, and *FADS1* rs174537 were associated with T2DM and CAD. The GG genotype of rs174537 led to an unfavorable FA status that is connected with T2DM and CAD [52]. Another study found that that minor alleles (T) of *FADS1* SNPs rs174537 and rs174546 were associated with lower plasma PA and AA levels in obese adolescents [49].

A Korean case–control study found that the minor allele (T) of *FADS1* rs174537 was associated with a lower risk of CAD that resulted from lower levels of plasma total cholesterol, LDL-C, a higher LDL particle size that was linked with oxidative stress markers (malondialdehyde, MDA; oxidatively modified LDL, ox-LDL, and urinary 8-*epi*-prostaglandin F_2α_ (8-*epi*-PGF_2α_)). The proportion of AA (20:4n-6) correlated with LDL-C, ox-LDL, MDA, and with urinary 8-*epi*-PGF_2α_. In controls, the G allele and the GG genotype were associated with opposite changes [37].

It has been proven that the G allele and the GG genotype of *FADS1* rs174537 are more prevalent among African Americans than among European Americans. This SNP is also associated with increased levels of AA, increased D5D (AA/DGLA ratio) and urinary concentrations of isoprostane F_2_ (F_2_-isoP) and F_3_-isoP; all markers of oxidative stress. African Americans are understood to be more effective converters of LA to AA [40,54]. In an Iranian study, minor alleles of *FADS1* rs174537 correlated with lower levels of LDL-C and a higher risk of T2DM [44]. *FADS1* (rs174545 and rs174546) and *FADS2* (rs174575) SNPs were connected with plasma lipids. Carriers of minor alleles had a lower plasma TC and LDL-C, whereas carriers of heterozygous alleles displayed higher TAG and lower HDL-C levels [31].

Minor alleles of various *FADS1* (rs174545, rs174546, rs174548, and rs174553) and *FADS2* (rs1535 and rs174583) genes have been associated with an increased risk of obesity in women with a lower BMI (less than 25.0 kg·m^−2^) [50]. While *FADS2* rs17457 minor allele carriers had a higher HOMA-IR index (connected with a higher level of DGLA), *FADS1* rs174546 predicted a lower risk of T2DM [27].

One study found lower levels of EPA and DPA in erythrocytes along with a decreased total n-3 FA in postmenopausal women. Female carriers of at least one minor allele of *FADS1* (rs174546) and *FADS2* (rs3834458) were associated with an unfavorable FA status contributing to MetS [12]. *FADS1* (rs174546) and *FADS2* (rs174601) were linked to CAD and ischemic stroke. Further, the T allele and the TT genotype were associated with these clinical conditions as well as with decreased apoA-I and HDL-C levels [28]. In a study involving individuals of Caucasian and Asian descent, minor alleles and genotypes of both *FADS1* (rs174546) and *FADS2* (rs174576) SNPs were connected with lower aggregate desaturase activity.

Finally, the minor allele of *FADS1* (rs174570) has been associated with decreased *FADS1* and *FADS2* expression levels, resulting from an epigenetic gene regulation (methylation) through CpG sites in *FADS1* and *FADS2* [76].

A limitation of this study was the relatively small number of participants and a cross-sectional design of the study. Another limitation of the current study was (from an analytical point of view) the absence of analyzed trans-FAs in plasma samples, the determination of the FA profiles as a relative percentage of the total esterified FAs of the plasma phospholipids, and, finally, the absence of simultaneously measured low-grade inflammation. Trans-FAs are supposed to have an important impact on the biochemical and clinical parameters of MetS [77]. The study utilized computational haplotype phasing as unrelated individuals were genotyped. Although based on a validated algorithm, which was shown to perform with high accuracy, it still represents a method of biostatistical inference with an inherent, though subtle, potential for errors. In humans, the biosynthesis of LC-PUFA requires delta-5 desaturase (D5D), D6D, and elongase 2 activities, which are encoded by the genes for *FADS1*, *FADS2*, and the elongation of very-long-chain fatty acids like 2 (*ELOVL2*). Neither elongase 2 activities nor genetic variants of *ELOVL2* were examined in the study.

The strength of this study was that the groups of participants were relatively homogenous. Patients with MetS were not treated either with lipid-lowering drugs, supplements containing n-3 PUFA/n-6 PUFA, or antioxidants. The implementation of a cluster analysis seems to be a novel approach. To date, the phenotyping of MetS based on a cluster analysis of FAs has not yet been carried out. We used the clustering approach to classify novel phenotypes of MetS. Our findings identified two distinct phenotypes of MetS through a cluster analysis according to plasma phospholipid FA composition. The current study represents a continuation of our previous studies/reports and contributes to the study of genetic factors, specifically *FADS* variants, to the fatty acid metabolism and pathogenesis of MetS phenotypes. Moreover, to our best knowledge, this was the first study to deal with several *FADS* variants in patients with MetS.

## 4. Materials and Methods

### 4.1. Patients

The sampling procedures used in this study were previously described in detail [9]. For the purpose of this paper, the study group remained unchanged. In brief, 354 subjects were enrolled as part of the study group, consisting of 188 (101 M/87F) controls (CON) and 166 MetS patients (98M/68F). MetS was diagnosed according to the International Diabetes Federation criteria [78]. The control group comprised 42 subjects (22M/20 F) without MetS components (MetSC) that were recruited from employees of the General University Hospital, as well as 146 probands (79M/67F) with at least one MetSC who failed to fulfil the diagnostic criteria for MetS. Of these, 69 probands had only one MetSC and 77 probands had two MetSCs.

Exclusion criteria for both groups were as follows: current antioxidant, lipid-lowering, and/or antidiabetic medication; excessive alcohol consumption (>30 g/day), hormonal replacement therapy, supplementation with polyunsaturated fatty acids (both of the n-3 and n-6 families), manifestation of cardiovascular and/or cerebrovascular diseases, type 1 diabetes mellitus, liver (except nonalcoholic fatty liver disease) and/or kidney diseases (creatinine >130 μmol/L), microalbuminuria (urinary albumin 30–300 mg/day), hypothyroidism, as well as recent serious infections and/or operations (both within the last six months), and malignancies.

Nutritional intake of the main dietary components, based on a standard 7-day dietary questionnaire, was assessed in all study subjects. Nutritional data were reappraised using the Nutrimaster SE software, Version 2.0 (2014).

Basal clinical and anthropometrical data, including body fat assessment, were examined using standard methods, as described previously [67]. The study was performed in adherence with the principles of the Declaration of Helsinki and approved by the Ethical Committee of the General University Hospital, Prague.

### 4.2. Laboratory Measurements

Total cholesterol (TC) in serum was analyzed using a commercially purchased enzymatic colorimetric test (CHOD-PAP) (BIO-LA-TEST Cholesterol 2500 kit; Pliva-Lachema, Czech Republic). Serum triacylglycerols (TAGs) were measured using a standard enzymatic colorimetric kit (GPO-PAP) (Pliva-Lachema). High-density lipoprotein cholesterol (HDL-C) concentration was measured in the supernatant of serum samples after selective precipitation of LDL-C using the BIO-LA-TEST HDL-Cholesterol kit (Pliva-Lachema) on a Cobas Mira analyzer (Roche, Switzerland).

To measure conjugated diene concentrations in precipitated LDL, a modified spectrophotometric method was used [68]. Levels of nonesterified fatty acids (NEFAs) were determined using an enzymatic colorimetric method (Randox Laboratories, UK). Apolipoprotein B concentration was analyzed nephelometrically using a standard kit (Beckman Coulter) on an image analyzer. Concentrations of insulin were measured using diagnostic sets and a modular analyzer (Roche Diagnostics, Indianapolis, IN, USA) by electrochemiluminescence (ECLIA).

Fatty acid patterns of the main plasma lipid classes were examined using analytical procedures described previously [79]. The homeostasis model assessment method, HOMA-IR, was used as an index of insulin resistance [80]. Desaturase activities were estimated using FA product/precursor ratios [58,67].

### 4.3. Clustering

Based on cluster analysis fatty acid (FA) patterns in plasma phospholipids independent of clinical and laboratory measures, all subjects were divided into two clusters (or phenotypes). As discussed in Section 2.1 in more detail, MetS was present mainly in cluster 1, while the CON probands were present mainly in cluster 2. In total, the whole study group was divided into four subgroups/clusters: MetS1 (*n* = 109), MetS2 (*n* = 57), CON1 (*n* = 71), and CON2 (*n* = 117).

Cluster analysis was performed in two steps. In the first step, the number of individual FAs in plasma phospholipids was reduced; in the second step, subjects were grouped into clusters. The number of individual FAs was reduced using linear discriminant analysis with stepwise forward variable selection based on Wilk’s lambda criterion. We used 22 of the initially analyzed FAs for all the probands to separate CON and MetS groups using linear discriminant analysis. This resulted in an overall 69.8% correct classification. The following variables subjected into the linear discriminant analysis were DHGLA (20:3n-6; F = 30.41), SA (18:0; F = 24.2), MA (14:0; F = 21.55), DHA (22:6n-3; F = 17.66), DPA (22:5n-3; F = 14.92), and LA (18:2n-6, F = 13.19) acids. These six individual fatty acids were included in the cluster analysis. On the selected six fatty acids, an agglomerative hierarchical clustering was applied using Ward’s minimum variance method with Euclidean measure [81]. This type of clustering minimizes the total within-cluster variance. Thus, the obtained clusters identified the groups of subjects with similar values of FAs selected with the discriminant analysis in the previous step.

### 4.4. Genetic Analyses

Genetic analyses were performed only in 330 subjects of the primary group (93.2%) of patients. The reasons for the reduction in the number of individuals analyzed were due to technical problems (lost or damaged blood samples in 8 cases) and disclaimers regarding informed consent for the genetic analysis (in 16 cases). The genetic analysis was performed in 180 subjects (96M/4F) from the CON group and 150 patients (83M/67F) from the MetS group.

Single-nucleotide nucleotide polymorphisms (SNPs) of two genes—fatty acid desaturase 1 (*FADS1*) and fatty acid desaturase 2 (*FADS2*) were analyzed in 330 participants. The numbers and sexes of the participants that took part in the genetic analyses across the various groups and clusters are given in the Supplementary Table S1. The three *FADS1* SNPs (rs174537 (extragenic), rs174545 (3′-UTR), and rs174546 (3′-UTR)) and five *FADS2* SNPs (rs174570, rs174575, rs174602, rs174589, and rs968567—all intronic) were selected for genotyping based on the following parameters: (i) their frequencies in the European Caucasian population [82], (ii) their established and implicated functional effects, as documented by previous genetic studies [14,16,22,36,37,38,52,83,84], and (iii) their positions along the *FADS1*/*FADS2* gene cluster [24]. All of the SNPs selected had a minor allele frequency (MAF) of > 0.1.

DNA isolation was performed according to standard desalting procedures [85]. The DNA concentration and purity were assessed (Nanodrop ND 1000, Thermo Scientific, Wilmington, DE, USA) as prerequisite parameters for subsequent methods. Polymerase chain reaction (PCR) conditions, forward and reverse primers, annealing temperatures, and restriction endonucleases are given in Table 8. Restriction products were directly separated by electrophoresis in 3% agarose gel and visualized in UV light after ethidium bromide staining. Fragment sizes were assessed using the NebCutter V2.0 program (http://tools.neb.com/NEBcutter2 (accessed on 15 May 2018)). For primer design, the Primer3 Input web application was used (http://tools.neb.com/NEBcutter2 (accessed on 15 May 2018)). Forward and reverse primers were further used for cycle sequencing to verify the results of the PCR restriction fragment length polymorphism (PCR-RFLP) method. In cases where the respective endonuclease or restrictive site was not found, direct DNA sequencing was employed [86]. Sequencing was performed using an automated DNA capillary sequencer (Model SEQ 8000, supplied by Beckman Coulter, Prague, Czech Republic).

The unphased genotype data of the whole cohort were entered into the Genotype Visualization and Algorithmic Tool (GEVALT) software, Version 2 [87]. Phasing, linkage disequilibrium analysis, and estimation of the haplotype block structure were determined using the Genotype Resolution and Block Identification using Likelihood (GERBIL) algorithm [88]. Multimarker haplotype associations were performed using the χ2-test implemented in the Haploview 4.2 software [89]. Multiple testing corrections were performed based on 1,000,000 permutations.

### 4.5. Statistical Analysis

The statistical analysis was performed using the R statistical software Version 4.1.3 [90], or with the STATISTICA^®^ software for Windows.

Categorical data were summarized using absolute and relative frequencies. Continuous data were expressed as mean and standard deviation or median and interquartile range (IQR, 25th–75th percentile) in cases of non-Gaussian distribution of data. The normality of the distribution was tested using the Shapiro–Wilks W test. Comparisons between the groups were determined using the independent *t*-test and the Wilcoxon test, respectively. *p*-values for both continuous and categorical data were adjusted for multiple comparisons using the Benjamini–Hochberg correction. Genotype distributions were tested for the Hardy–Weinberg equilibrium (HWE) (with d.f. = N-2). Pearson’s χ^2^-test was employed to test the differences in the distribution of genotype frequencies in both groups (Yates’ correction for small numbers in 2 × 2 tables). Statistical significance was defined as *p* < 0.05.

## 5. Conclusions

In summary, the current study demonstrated significant associations between a number of *FADS1*, *FADS2* polymorphisms, and two MetS phenotypes identified by a cluster analysis of plasma FA profiles. The first phenotype had a more harmful clinical and metabolic profile (increased waist circumference, increase insulin resistance and oxidative stress) than the second phenotype. The *FADS1* (rs174537, rs174545, and rs174546) and *FADS2* (174570 and 174602) polymorphisms, along with the two inferred haplotypes (GGGCC; TCACT) described in this study, were found to be implicated in the heterogeneity of MetS.

The findings of this study could contribute (from a gnoseological point of view) to the knowledge of the regulation of FA metabolism and the pathogenesis of MetS; from a clinical point of view, it could be a contribution to the personalized management of MetS.

The subjects carrying reference alleles of *FADS1* (*FADS2*, respectively) having a higher conversion capacity towards PUFA n-6 products might benefit from PUFA n-3 supplementation (or intervention).

## Figures and Tables

**Table 1:** Clinical and biochemical characteristics of studied groups.

Parameter	MetS	CON
Number of persons	166	188
Gender (M/F)	98/68 ^a^	101/87^NS b^
Age (years)	55.2 ± 10.6	54.5 ± 11.9
Body weight (kg)	88.0/19.9 ***	75.7/17.2
BMI (kg·m^−2^)	29.3/4.3 ***	26.0/4.9
Waist circumference (cm)	103 ± 10 ***	91 ± 12
Systolic BP (mm Hg)	140/20 ***	130/20
Diastolic BP (mm Hg)	90/14 ***	80/10
Relative fat mass (%)	35.4/11.1 ***	30.0/11.2
Fat mass (kg)	28.5/9.6 ***	21.9/10.3
Glucose (mmol/L)	5.60/1.5 ***	4.90/0.7
Insulin (mU/L)	10.70/7.24 ***	7.70/5.56
HOMA-IR (ratio)	2.593/2.142 ***	1.622/1.203
TC (mmol/L)	6.29/1.68 *	5.88/1.98
TAG (mmol/L)	2.69/2.09 ***	1.40/0.83
HDL-C (mmol/L)	1.23/050 ***	1.50/0.54
NEFA (mmol/L)	0.600/0.530 **	0.530/0.360
Apo B (g/L)	1.33/041 ***	1.17/0.52
CD-LDL (μmol/L)	66.7/23.7 ***	56.4/22.9

Data are in mean ± SD or median/IQR format; ^a^ number of subjects according to gender (%) in individual phenotypes of MetS. *p* values were adjusted for multiple comparisons using Benjamini–Hochberg corrections: * *p* < 0.05, ** *p* < 0.01, and *** *p* < 0.001. ^b^ Pearson χ^2^ test for testing differences in categorical data (Yates’ χ^2^ test for small numbers): * *p* < 0.05. Abbreviations: MetS—metabolic syndrome; M—males; F—females; BMI—body mass index; BP—blood pressure; NEFA—nonesterified fatty acids; CD-LDL—conjugated dienes in LDL; CON—control group; MetS—metabolic syndrome; TC—total cholesterol; TAG—triacylglycerols; LDL—low-density lipoproteins; HDL—high-density lipoproteins; Apo—apolipoprotein; HOMA-IR—homeostasis model assessment for insulin resistance (f-insulin (μU/mL) × f-glucose (mmol/L)/22.5); IQR—interquartile range; NS – not significant.

**Table 2:** Clinical and biochemical characteristics of metabolic syndrome according to cluster analysis.

Parameter	MetS—Cluster 1	MetS—Cluster 2
Number of persons	109	57
Gender (M/F)	67/42 ^a^	31/26
Age (years)	54.6 ± 11.1	56.3 ± 9.5
Body weight (kg)	90.0/19.0	85.8/20.3
BMI (kg·m^−2^)	29.7/4.3	28.4/4.4
Waist circumference (cm)	105 ± 11 *	101 ± 9
Systolic BP (mm Hg)	140/20	140/20
Diastolic BP (mm Hg)	90/10	89/10
Relative fat mass (%)	33.7/10.3	37.5/11.6
Fat mass (kg)	28.5/8.9	28.6/11.2
Glucose (mmol/L)	5.7/1.8	5.3/1.1
Insulin (mU/L)	11.75/7.17	9.40/5.83
HOMA-IR (ratio)	3.03/2.30 *	2.07/1.94
TC (mmol/L)	6.40/1.89	6.10/1.46
TAG (mmol/L)	2.86/3.09	2.43/1.60
HDL-C (mmol/L)	1.21/0.48	1.24/0.48
NEFA (mmol/L)	0.690/0.730 ***	0.440/0.398
Apo B (g/L)	1.32/0.44	1.36/0.38
CD-LDL (μmol/L)	70.9/34.9 *	61.0/19.7

For legend and abbreviations, see Table 1.

**Table 3:** Clinical and biochemical characteristics of the control group based on cluster analysis.

Parameter	CON—Cluster 1	CON—Cluster 2
Number of persons	71	117
Gender (M/F)	43/28	58/59
Age (years)	53.8 ± 10.7	54.9 ± 12.6
Body weight (kg)	80.6/23.8 **	73.7/15.7
BMI (kg·m^−2^)	26.7/5.1 *	25.3/4.5
Waist circumference (cm)	95.5 ± 12.3 ***	88.9 ± 10.6
Systolic BP (mm Hg)	130/20	130/20
Diastolic BP (mm Hg)	80/10	80/5
Relative fat mass (%)	31.2/11.3	29.5/11.4
Fat mass (kg)	24.0/10.9	20.8/9.7
Glucose (mmol/L)	5.00/0.60	4.90/0.80
Insulin (mU/L)	8.59/6.00	7.43/5.3.0
HOMA-IR (ratio)	1.820/1.412 *	1.568/1.226
TC (mmol/L)	6.09/2.21	5.73/1.94
TAG (mmol/L)	1.57/1.10 **++	1.27/0.7
HDL-C (mmol/L)	1.49/0.41	1.52/0.58
NEFA (mmol/L)	0.535/0.403	0.520/0.300
Apo B (g/L)	1.22/0.55	1.13/0.46
CD-LDL (μmol/L)	62.3/23.9	55.5/24.8

For legend and abbreviations, see Table 1; ANCOVA (adjusted with body weight as the covariate): *p <* 0.05, ++ *p <* 0.01.

**Table 4:** Plasma phospholipid fatty acid composition of the analyzed groups.

Fatty Acid	MetS (*n* = 166)	CON (*n* = 188)
14:0 ^a^	0.266/0.104	0.276/0.105
16:0	29.683/1.974	29.364/1.755
16:1n-9	0.102/0.038	0.111/0.043
16:1n-7	0.593/0.243 **	0.522/0.197
18:0	14.43 ± 1.28 ***	13.84 ± 1.14
18:1n-9	9.850/1.971	9.795/2.050
18:1n-7	1.490/0.422	1.549/0.372
18:2n-6	21.94 ± 0.16 ***	23.54 ± 3.00
18:3n-6	0.084/0.052	0.076/0.046
18:3n-3	0.191/0.081	0.209/0.096
20:2n-6	0.401/0.138	0.398/0.141
20:3n-6	3.351/0.803 ***	3.011/0.764
20:4n-6	10.99 ± 2.05	10.91 ± 1.83
20:5n-3	0.943/0.497	0.924/0.483
22:4n-6	0.310/0.092	0.312/0.078
22:5n-6	0.193/0.076	0.194/0.063
22:5n-3	0.892/0.200	0.891/0.207
22:6n-3	3.441/1.250	3.243/1.157
∑satur	44.368/1.640 ***	43.552/1.955
∑MFA	12.197/2.407	12.039/2.614
∑n-6	37.251/3.772 ***	38.641/3.285
∑n-3	5.524/1.885	5.308/1.623
D9D 16 (16:1n-7/16:0)	0.020/0.008 **	0.018/0.007
D9D 18 (18:1n-9/18:0)	0.678/0.171	0.709/0.154
D6D n-6 (18:3n-6/18:2n-6)	0.004/0.003 *	0.003/0.002
D5D n-6 (20:4n-6/20:3n-6)	3.117/1.251 **	3.605/1.318

Data are in mean ± SD or media/interquartile range; ^a^ shorthand notation of fatty acids: carbon number: double bond number, n-position of carbon with first double bond from methyl end; *p* values were adjusted for multiple comparisons using Benjamini–Hochberg corrections: * *p* < 0.05, ** *p* < 0.01, and *** *p* < 0.001. Abbreviations: ∑satur—sum of saturated fatty acids; ∑MFA—sum of monounsaturated fatty acids; ∑n-6—sum of n-6 polyunsaturated fatty acids; ∑n-3—sum of n-3 polyunsaturated fatty acids.

**Table 5:** Plasma phospholipid fatty acid composition of metabolic syndrome based on cluster analysis.

Fatty Acid	MetS—Cluster 1 (*n* = 109)	MetS—Cluster 2 (*n* = 57)
14:0 ^a^	0.268/0.110	0.264/0.105
16:0	29.752/1.903	29.091/2.072
16:1n-9	0.105/0.041	0.098/0.037
16:1n-7	0.634/0.284 ***	0.484/0.183
18:0	14.59 ± 1.34	14.16 ± 1.12
18:1n-9	10.154/1.945 ***	8.930/1.453
18:1n-7	1.556/0.467 **	1.382/0.291
18:2n-6	20.17 ± 2.07 ***	25.31 ± 1.88
18:3n-6	0.089/0.053 *	0.074/0.036
18:3n-3	0.198/0.081	0.186/0.078
20:2n-6	0.408/0.149	0.381/0.121
20:3n-6	3.363/0.708 *	3.036/0.893
20:4n-6	11.34 ± 2.03 **	10.34 ± 1.95
20:5n-3	1.091/0.492 ***	0.801/0.284
22:4n-6	0.312/0.103 *	0.284/0.098
22:5n-6	0.199/0.075	0.181/0.072
22:5n-3	0.909/0.192 ***	0.818/0.164
22:6n-3	3.574/1.251 **	3.018/0.973
∑satur	44.722/1.698 ***	43.665/.1,225
∑mono	12.812/2.555 ***	11.034/1.957
∑n-6	36.428/3.163 ***	39.821/3.098
∑n-3	5.817/1.497 ***	4.931/1.101
D9D 16 (16:1n-7/16:0)	0.021/0.010 ***	0.016/0.005
D9D 18 (18:1n-9/18:0)	0.736/0.175 **	0.646/0.150
D6D n-6 (18:3n-6/18:2n-6)	0.005/0.003 ***	0.003/0.002
D5D n-6 (20:4n-6/20:3n-6)	3.383/1.182	3.214/1.595

For legend and abbreviations, see Table 4.

**Table 6:** Numbers and frequencies of allele and genotype variants for *FADS1*–*FADS2* genes across studied groups.

Polymorphism	Group (Size)	A	a	AA	Aa	aa
***FADS1*** **rs174537** ^a^	MetS (150)	G 204 (68.0)	T 96 (32.0)	GG 70 (46.7)	GT 64 (42.7)	TT 16 (10.6)
	CON (180)	G 239 (66.4)	T 121 (33.6)	GG 74 (41.1)	GT 91 (50.6)	TT 15 (8.3)
	CON1 (68)	G 99 (72.8)	T 37 (27.2)	GG 34 (50.0)	GT 31 (45.6)	TT 3 (4.4)
	CON2 (112)	G 140 (62.5)	T 84 (37.5)	GG 40 (35.7)	GT 60 (53.6)	TT 12 (10.7)
***FADS2*** **rs174570**	MetS (150)	C 257 (85.7)	T 43 (14.3)	CC 110 (73.3)	CT 37 (24.7)	TT 3 (2.0)
	CON (180)	C 314 (87.2)	T 46 (12.8)	CC 135 (75.0)	CT 44 (24.4)	TT 1 (0.6)
	CON1 (68)	C 124 (91.2)	T 12 (8.8)	CC 56 (82.4)	CT 12 (17.6)	TT 0 (0)
	CON2 (112)	C 190 (84.8)	T 34 (15.2)	CC 79 (70.5)	CT 32 (28.6)	TT 1 0.9)
***FADS2*** **rs174575**	MetS (150)	C 234 (78.0)	G 66 (22)	CC 90 (60.0)	CG 54 (36.0)	GG 6 (4.0)
	CON (180)	C 264 (73.3)	G 96 (26.7)	CC 95 (52.8)	CG 74 (41.1)	GG 11 (6.1)
	CON1 (68)	C 105 (77.2)	G 31 (22.8)	CC 41 (60.3)	CG 23 (33.8)	GG 4 (5.9)
	CON2 (112)	C 159 (71.0)	G 65 (29.0)	CC 54 (48.2)	CG 51 (45.5)	GG 7 (6.3)
***FADS2*** **rs174602**	MetS (150)	T 247 (82.3)	C 53 (17.7)	TT 102 (68.0)	TC 43 (28.7)	CC 5 (3.3)
	CON (180)	T 298 (82.8)	C 62 (17.2)	TT 122 (67.8)	TC 54 (30.0)	CC 4 2.2)
	CON1 (68)	T 118 (86.8)	C18 (13.2)	TT 51 (75.0)	TC 16 (23.5)	CC 1 (1.5)
	CON2 (112)	T 180 (80.4)	C 44 (19.6)	TT 71 (63.4)	TC 38 33.9)	CC 3 (2.7)
***FADS2*** **rs174589**	MetS (150)	C 244 (81.3)	G 56 (18.7)	CC 99 (66.0)	CG 46 (30.7)	GG 5 (3.3)
	CON (180)	C 295 (81.9)	G 65 (18.1)	CC 117 (65.0)	CG 61 (33.9)	GG 2 (1.1)
	CON1 (68)	C 118 (86.8)	G 18 (13.2)	CC 50 (73.5)	CG 18 (26.5)	GG 0 (0)
	CON2 (112)	C 177 (79.0)	G 47 (21.0)	CC 67 (59.8)	CG 43 (38.4)	GG 2 (1.8)
***FADS2*** **rs968567**	MetS (150)	C 259 (86.3)	T 41 (13.7)	CC 111 (74.0)	CT 37 (24.7)	TT 2 (1.3)
	CON (180)	C 300 (83.3)	T 60 (16.7)	CC 123 (68.3)	CT 54 (30.0)	TT 3 (1.7)
	CON1 (68)	C 117 (86.0)	T 19 (14.0)	CC 50 (73.5)	CT 17 (25.0)	TT 1 (1.5)
	CON2 (112)	C 183 (81.7)	T 41 (18.3)	CC 73 (65.2)	CT 37 (33.0)	TT 2 (1.8)

^a/^Numbers and frequencies of alleles and genotypes of *FADS1* rs174547 and *FADS1* rs174546 are identical to those of *FADS1* rs174537. Abbreviations: MetS—metabolic syndrome; CON—control group; CON1—cluster 1 of CON; CON2—cluster 2 of CON. A comparison between MetS1 and MetS2 group is presented in detail in Table 7. A—major allele; a—minor allele; *FADS1* rs174537 (G/T) was in the complete linkage disequilibrium (LD) (r^2^ = 1.0) with *FADS1* SNPs rs 174545 (G/C) and rs174546 (G/A) (not shown).

**Table 7:** Genotype and allele frequencies of studied *FADS* polymorphisms in MetS clusters 1 and 2.

Polymorphism	MetS—Cluster 1 (*n*= 94)		MetS—Cluster 2 (*n* = 56)		χ^2^ Test ^a^
	Number	%	Number	%	
*FADS1* (rs174537 G/T) ^e^					
GG	52	55.3	18	32.1	χ^2^ = 14.039 ^b^ *p* = 0.0024 ^d^
GT	38	40.4	26	46.5	
TT	4	4.3	12	21.4	
G	142	75.5	62	55.4	χ^2^ = 12.218 ^c^ *p* = 0.0024
T	46	24.5	50	44.6	
*FADS2* (rs 174570 C/T)					
CC	76	80.9	34	60.7	χ^2^ = 10.084 *p* = 0.014
CT	18	19.1	19	33.9	
TT	0	0	3	5.4	
C	170	90.4	87	77.7	χ^2^ = 8.279 *p* = 0.009
T	18	9.6	25	22.3	
*FADS2* (rs174575 C/G)					
CC	62	66.0	28	50.0	χ^2^ = 4.863 *p* = 0.105
CG	30	31.9	24	42.9	
GG	2	2.1	4	7.1	
C	154	81.9	80	71.4	χ^2^ = 3.907 *p* = 0.064
G	34	18.1	32	28.6	
*FADS2* (rs174602 T/C)					
TT	70	74.5	32	57.2	χ^2^ = 6.988 *p* = 0.048
TC	23	24.5	20	35.7	
CC	1	1.0	4	7.1	
T	163	86.7	84	75.0	χ^2^ = 5.828 *p* = 0.0284
C	25	13.3	28	25.0	
*FADS2* (rs174589 C/G)					
CC	67	71.3	32	57.2	χ^2^ = 5.695 *p* = 0.0713
CG	26	27.7	20	35.7	
GG	1	1.0	4	7.1	
C	160	85.1	84	75.0	χ^2^ = 4.080 *p* = 0.0625
G	28	14.9	28	25.0	
*FADS2* (rs968567 C/T)					
CC	73	77.7	38	67.9	χ^2^ = 4.365 *p* = 0.1205
CT	21	22.3	16	28.6	
TT	0	0	2	3.5	
C	167	88.8	92	82.1	χ^2^ = 2.123 *p* = 0.145
T	21	11.2	20	17.9	

^a^/Pearson χ^2^ test (Yates’ correction for low numbers); ^b/^genotype; ^c/^allele; ^d/^Benjamini–Hochberg correction for multiple comparisons; ^e/^*FADS1* rs174537 (G/T) was in the complete linkage disequilibrium (LD) (r^2^ = 1.0) with the *FADS1* SNPs rs 174545 (G/C) and rs174546 (G/A) (not shown). Abbreviations: MetS—metabolic syndrome; MetS1–MetS cluster 1; MetS2 –MetS cluster 2. A—major allele; a—minor allele.

**Table 8:** Analytical conditions for detection of polymorphisms in the *FADS1* and *FADS2* genes.

Genes	Polymorphisms ^1^	Forward Primers 5′→ 3′ Reverse Primers 5′→ 3′	Annealing (°C)	Methods RFLP, Direct Sequencing	
				Restrictase	Sequencing
*FADS1*	rs174537 G > T	caggggagagaggtggagta aggtctgtctggctgtctcc	59.3	AvaII	
	rs174545 G > C	ccatcctcatttgcaaacct cagcagcctaaggcagacat	60.2	CviKI-1	
	rs174546 G > A	gccttaacctcactgctcca aggctttatgtccccaaacc	60.3	BsaJI	
*FADS2*	rs174570 C > T	agaggcaaggagggaagaaa cgggcctacacagcttagag	60.2	BsaBI	
	rs174575 C > G	ctcagaagttggggcttgag actccaagggagcagacaga	60.0	BlpI	Direct sequencing
	rs174602 T > C	aggaaagggacagtggtgtg ctggtgattgtagggcaggt	60.0	BtsCI	
	rs174589 C > G	gccaagcctaacatcttcca ctaggcttccttccctgctc	60.3	-	Direct sequencing
	rs968567 C > T, A, G	aagatcctcctgggccaat gctatggacttttgcctcca	60.5	SacI	Direct sequencing

^1^ Gene polymorphisms and allelic variants were denominated according to the National Center of Biotechnology Information (http://www.ncbi.nlm.nih.gov (accessed on 16 August 2017)). Abbreviations: A—adenine; C—cytosine; G—guanine; T—thymine.

## Data Availability

All the data supporting the findings of this study are included in this article and the Supplementary Materials.

## Supplementary Materials

The following are available online at https://www.mdpi.com/article/10.3390/metabo12060568/s1, Table S1: clinical and biochemical characteristics of participants in MetS1 and CON1 groups, Table S2: clinical and biochemical characteristics of participants in MetS2 and CON2 groups, Table S3: plasma phospholipid fatty acid composition of control groups based on cluster analysis, Table S4: plasma phospholipids fatty acid composition of cluster 1 in metabolic syndrome and control groups, Table S5: plasma phospholipids fatty acid composition of cluster 2 in metabolic syndrome and control groups, Table S6: reference and alternative allele frequencies according to NCBI Allele Frequency Aggregator (ALFA) Release 2.
