# Supplementary material for: FADS Polymorphisms Affect the Clinical and Biochemical Phenotypes of Metabolic Syndrome
Source: Metabolites. 2022 Jun 20;12(6):568. doi: 10.3390/metabo12060568 (PMC9228863; doi:10.3390/metabo12060568)
Supplement: Supplementary file 1 [file metabolites-12-00568-s001.zip › Suppl Table S1 MetS1 CON1 F.pdf]

Supplementary Table S1. Clinical and biochemical characteristics of participants in MetS1 and CON1 groups

	**MetS 1**	**CON 1**
Number of persons	109	71
Gender (M/F)	67/42	43/28
Age (years)	54.6 ± 11.1	53.8 ± 10.7
Body weight (kg)	90.0/19.0**	80.6/23.8
BMI ($kg.m^{-2}$)	29.7/4.3***+	26.7/5.1
Waist circumference (cm)	104.3 ± 10.6*** ++	95.5 ± 12.2
Systolic BP (mm Hg)	140/20*** +++	130/20
Diastolic BP (mm Hg)	90/15*** +++	80/10
Relative fat mass (%)	33.6/10.3*+	31.2/11.3
Fat mass (kg)	28.5/8.9***+	24.0/10.9
Glucose (mmol/l)	5.70/1.80*** +++	5.00/0.60
Insulin (mU/l)	11.75/7.17** +	8.59/6.00
HOMA-IR (ratio)	3.033/2.301*** ++	1.820/1.412
TC (mmol/l)	6.40/1.89	6.09/2.20
TAG (mmol/l)	2,86/3.09*** +++	1.57/1.03
HDL-C (mmol/l)	1.21/0.48*** ++	1.49/0.41
NEFA (mmol/l)	0.690/0.790** ++	0.535/0.430
Apo B (g/l)	1.32/0.44	1.22/0.550
CD-LDL (μmol/l)	70.9/34.9**++	60.3/23.9

Data are in mean ± SD or median/IQR format; a number of subjects according to gender (%) in individual phenotypes of MetS. P values were adjusted for multiple comparisons using Benjamini-Hochberg corrections: * P < 0.05, ** P < 0.01, *** P < 0.001. b Pearson $\chi2$ test for testing differences of categorical data (Yates´ $\chi2$ test for small numbers): * P < 0.05. Abbreviations: MetS – metabolic syndrome, M – males, F – females, BMI – body mass index; BP – blood pressure; NEFA – nonesterified fatty acids; CD-LDL – conjugated dienes in LDL; CON – control group, MetS – metabolic syndrome, TC – total cholesterol; TAG – triacylglycerols; LDL – low density lipoproteins; HDL – high density lipoproteins; Apo – apolipoprotein, HOMA-IR - homeostasis model assessment for insulin resistance (f-insulin (μU/ml) x f-glucose (mmol/l) / 22.5); IQR – interquartile range; ANCOVA (adjusted with body weight as covariate): + P<0.05; ++ P<0.01; +++ P<0.001
