# Supplementary material for: FADS Polymorphisms Affect the Clinical and Biochemical Phenotypes of Metabolic Syndrome
Source: Metabolites. 2022 Jun 20;12(6):568. doi: 10.3390/metabo12060568 (PMC9228863; doi:10.3390/metabo12060568)
Supplement: Supplementary file 1 [file metabolites-12-00568-s001.zip › Suppl Table S2 Met2 CON2 F.pdf]

Supplementary Table S2. Clinical and biochemical characteristics of participants in MetS2 and CON2 groups

	**MetS2**	**CON 2**
Number of persons	57	117
Gender (M/F)	31/26	58/59
Age (years)	56.3 ± 9.5	54.9 ± 12.6
Body weight (kg)	85.8/20.3**	80.6/23.8
BMI ($kg·m^{-2}$)	28.4/4.3***	25.3/4.5
Waist circumference (cm)	101 ± 9*** ++	89 ± 11
Systolic BP (mm Hg)	140/20*** +++	130/20
Diastolic BP (mm Hg)	90/10*** +++	80/5
Relative fat mass (%)	37.5/11.6**++	29.5/11.4
Fat mass (kg)	28.6/11.2***++	20.8/10.9
Glucose (mmol/l)	5.30/1.10*** +	4.90/0.80
Insulin (mU/l)	9.40/5.83**	7.43/5.30
HOMA-IR (ratio)	3.073/1.943*** +	1.568/1.226
TC (mmol/l)	6.10/1.46	5.73/1.94
TAG (mmol/l)	2.34/1.60*** +++	1.27/0.70
HDL-C (mmol/l)	1.24/0.48** +	1.52/0.58
NEFA (mmol/l)	0.440/0.397	0.520/0.300
Apo B (g/l)	1.36/0.38**+	1.13/0.45
CD-LDL (µmol/l)	61.0/19.7	55.5/24.8

Legend and abbreviations: see Supplementary Table 1; ANCOVA (adjusted with body weight as covariate): + P<0.05; ++ P<0.01; +++ P<0.001
