# Supplementary material for: FADS Polymorphisms Affect the Clinical and Biochemical Phenotypes of Metabolic Syndrome
Source: Metabolites. 2022 Jun 20;12(6):568. doi: 10.3390/metabo12060568 (PMC9228863; doi:10.3390/metabo12060568)
Supplement: Supplementary file 1 [file metabolites-12-00568-s001.zip › Suppl Table S3 FA CON1 CON2.pdf]

Supplementary Table S3 Plasma phospholipid fatty acid composition of control group according to cluster analysis.

**Fatty acid**	**CON – Cluster 1 (n=71)**	**CON – Cluster 2 (n=117)**
14:0[a]	0.283/0.119	0.275/0.096
16:0	30.016/2.271**	29.274/1.365
16:1n-9	0.113/0.048	0.109/0.042
16:1n-7	0.598/0.286***	0.492/0.182
18:0	14.23 ± 1.25**	13.61 ± 1.01
18:1n-9	10.144/2.300*	9.574/1.914
18:1n-7	1.530/0.493	1.560/0.310
18:2n-6	20.51 ± 1.82***	25.38 ± 1.87
18:3n-6	0.089/0.050**	0.070/0.037
18:3n-3	0.198/0.098	0.209/0.089
20:2n-6	0.386/0.175	0.401/0.129
20:3n-6	3.136/0.847***	2.894/0.685
20:4n-6	11.66 ± 1.91***	10.46 ± 1.62
20:5n-3	1.132/0.566***	0.836/0.387
22:4n-6	0.334/0.084***	0.301/0.069
22:5n-6	0.203/0.059**	0.189/0.060
22:5n-3	0.958/0.209***	0.840/0.172
22:6n-3	3.584/1.118**	3.089/0.714
Σsatur	44.481/1.163***	43.099/1.339
Σmono	12.596/2.685**	11.741/2.200
Σn-6	36.697/3.363***	39.642/2.897
Σn-3	5.768/1.503***	4.886/1.422
D9D 16 (16:1n-7/16:0)	0.020/0.010***	0.017/0.006
D9D 18 (18:1n-9/18:0)	0.729/0.164	0.708/0.141
D6D n-6 (18:3n-6/18:2n-6)	0.004/0.003***	0.003/0.002
D5D n-6 (20:4n-6/20:3n-6)	3.605/1.361	3.601/1.273

Data are in mean ± SD or media/interquartile range; [a] shorthand notation of fatty acids: carbon number:double bond number, n-position of carbon with first double bond from methyl end; P values were adjusted for multiple comparisons using Benjamini-Hochberg corrections: * $P < 0.05$, ** $P < 0.01$, *** $P < 0.001$. Abbreviations: Σsatur – sum of saturated fatty acids, Σmono – sum of monosaturated fatty acids, Σn-6 – sum of n-6 polyunsaturated fatty acids, Σn-3 – sum of n-3 polyunsaturated fatty acids,
