# Supplementary material for: FADS Polymorphisms Affect the Clinical and Biochemical Phenotypes of Metabolic Syndrome
Source: Metabolites. 2022 Jun 20;12(6):568. doi: 10.3390/metabo12060568 (PMC9228863; doi:10.3390/metabo12060568)
Supplement: Supplementary file 1 [file metabolites-12-00568-s001.zip › Suppl Table S4 FA MetS1 CON1.pdf]

Supplementary Table S4 Plasma phospholipid fatty acid composition in cluster 1 in metabolic syndrome and control groups.

Fatty acid	MetS – Cluster 1 (n=109)	CON – Cluster 1 (n=71)
14:0[a]	0.268/0.110	0.283/0.119
16:0	29.752/1.903	30.016/2.271
16:1n-9	0.105/0.041	0.113/0.048
16:1n-7	0.634/0.284	0.598/0.286
18:0	14.59 ± 1.34	14.23 ± 1.25
18:1n-9	10.154/1.945	10.144/2.300
18:1n-7	1.556/0.467	1.530/0.493
18:2n-6	20.17 ± 2.07	20.51 ± 1.82
18:3n-6	0.089/0.053	0.089/0.050
18:3n-3	0.198/0.081	0.198/0.098
20:2n-6	0.408/0.149	0.386/0.175
20:3n-6	3.363/0.708*	3.136/0.847
20:4n-6	11.34 ± 2.03	11.66 ± 1.91
20:5n-3	1.091/0.492	1.132/0.566
22:4n-6	0.312/0.103	0.334/0.084
22:5n-6	0.199/0.075	0.203/0.059
22:5n-3	0.909/0.192	0.958/0.209
22:6n-3	3.574/1.251	3.584/1.118
Σsatur	44.722/1.698	44.481/1.163
Σmono	12.812/2.555	12.596/2.685
Σn-6	36.428/3.163	36.697/3.363
Σn-3	5.817/1.497	5.768/1.503
D9D 16 (16:1n-7/16:0)	0.021/0.010	0.020/0.010
D9D 18 (18:1n-9/18:0)	0.736/0.175	0.729/0.164
D6D n-6 (18:3n-6/18:2n-6)	0.005/0.003	0.004/0.003
D5D n-6 (20:4n-6/20:3n-6)	3.383/1.182*	3.605/1.361

See Supplementary Table S3 for abbreviations and legend
