# Supplementary material for: FADS Polymorphisms Affect the Clinical and Biochemical Phenotypes of Metabolic Syndrome
Source: Metabolites. 2022 Jun 20;12(6):568. doi: 10.3390/metabo12060568 (PMC9228863; doi:10.3390/metabo12060568)
Supplement: Supplementary file 1 [file metabolites-12-00568-s001.zip › Suppl Table S5 FA MetS2 CON2.pdf]

Supplementary Table S5 Plasma phospholipid fatty acid composition of cluster 2 in metabolic syndrome and control groups.

Fatty acid	MetS – Cluster 2 (n=57)	CON – Cluster 2 (n=117)
14:0[a]	0.264/0.105	0.275/0.096
16:0	29.091/2.072	29.274/1.365
16:1n-9	0.098/0.037	0.109/0.042
16:1n-7	0.484/0.183	0.492/0.182
18:0	14.16 ± 1.12**	13.61 ± 1.01
18:1n-9	8.930/1.453**+	9.574/1.914
18:1n-7	1.382/0.291	1.560/0.310
18:2n-6	25.31 ± 1.88	25.38 ± 1.87
18:3n-6	0.074/0.036	0.070/0.037
18:3n-3	0.186/0.078	0.209/0.089
20:2n-6	0.381/0.121	0.401/0.129
20:3n-6	3.036/0.893**+	2.894/0.685
20:4n-6	10.34 ± 1.95	10.46 ± 1.62
20:5n-3	0.801/0.284	0.836/0.387
22:4n-6	0.284/0.098	0.301/0.069
22:5n-6	0.181/0.072	0.189/0.060
22:5n-3	0.818/0.164	0.840/0.172
22:6n-3	3.018/0.973	3.089/0.714
Σsatur	43.665/.1,225***+	43.099/1.339
Σmono	11.034/1.957**+	11.741/2.200
Σn-6	39.821/3.098	39.642/2.897
Σn-3	4.931/1.101	4.886/1.422
D9D 16 (16:1n-7/16:0)	0.016/0.005	0.017/0.006
D9D 18 (18:1n-9/18:0)	0.646/0.150**+	0.708/0.141
D6D n-6 (18:3n-6/18:2n-6)	0.003/0.002	0.003/0.002
D5D n-6 (20:4n-6/20:3n-6)	3.214/1.595*	3.601/1.273

See Supplementary Table S3 for abbreviations and legend
