# Supplementary material for: FADS Polymorphisms Affect the Clinical and Biochemical Phenotypes of Metabolic Syndrome
Source: Metabolites. 2022 Jun 20;12(6):568. doi: 10.3390/metabo12060568 (PMC9228863; doi:10.3390/metabo12060568)
Supplement: Supplementary file 1 [file metabolites-12-00568-s001.zip › Suppl Table S6 allele freq FADS SNPs Evrope vs study.pdf]

Supplementary Table S6 Reference and alternative allele frequencies according to NCBI Allele Frequency Aggregator (ALFA) Release 2

SNP FADS/rs	Reference allele (European populations)	Alternative allele (European populations)	Reference allele (Current Study)	Alternative allele (Current Study)
(*FADS1*) rs174537 G>C/T	0.66585	0.33415	0.6712	0.3288
FADS1 rs174545 C>A/G	0.65904	0.34096	0.6712	0.3288
FADS1 rs174546 C>T	0.674846	0.325154	0.6712	0.3288
FADS2 rs174570 C>G/T	0.873882	T= 0.126118; G=0	0.8652	0.1348
FADS2 rs174575 C>G	0.75833	0.24167	0.7545	0.2455
FADS2 rs174602 T>C	0.79907	0.20093	0.8258	0.1742
FADS2 rs174589 C>G/T	0.87202	G=0.12798; T=0	0.8167	0.1833
FADS2 rs968567 C>T	0.83671	0.16329	0.8470	0.1530

legend FADS – fatty acid desaturase, SNP – single nucleotide polymorphism
